# Erythema; Produced by Local Irritation

**Published:** 1861-07

**Authors:** 


IV. *Erythema; produced by local irritation.*—P. C., a native of England, æt 43, was admitted June 19. Had syphilis 16 years ago, for which he was treated successfully in the Dreadnaught Hospital ship. Is of a robust constitution, plethoric habit, and has always enjoyed good health. Two years ago he suffered three weeks from an attack similar to the present one, produced by work in a harvest field, and again last October, from poisoning by the ivy vine when fishing. His present difficulty he attributes to the irritation of dust whilst loading a vessel with grain, the 9th of June.

The face, neck, arms and scrotum presented, upon admission, a shining, red, inflamed and swollen appearance, more marked where the integuments are thin, attended with itching and a painful sense of fulness. The face and neck were indeed of a dusky purplish color, closely resembling erysipelas, except in the distension of the subcutaneous areolar tissue. Upon the scrotum, portions of the neck, bend of the arms, and the eyelids, the skin was cracked, and the lines formed a net-work, more or less raised by the edges of the ruptured dermis being inflamed and swollen; from these fissures exuded a thin, ichorous discharge of serous fluid, which formed crusts, and would subsequently rub off with scales of the epidermis, giving the eruption in these places the appearance of eczema. In other places the disease was less severe, for instance, upon the thicker integuments of the outside of the arms, occurring in patches somewhat papulous, terminating by exfoliation of the epidermis in thin scales or furfuraceous desquamation.

June 29.—Patient was discharged cured.

The treatment was by tne use of saline laxatives, and the topical use of a weak solution of sulphuret of potash; at night, a chloroform ointment was used to allay the itching.

